# Late Diagnosis of Klinefelter Syndrome: Overcoming Phenotypic Variability and Diagnostic Oversights

**DOI:** 10.1155/crie/6399278

**Published:** 2025-08-29

**Authors:** Amna Kamran, Chinelo Okigbo

**Affiliations:** Division of Endocrine and Metabolic Disorders, Department of Medicine, Macon and Joan Brock Virginia Health Sciences at Old Dominion University, Norfolk, Virginia, USA

## Abstract

We report a case of Klinefelter syndrome (KS) diagnosed in adulthood, emphasizing the impact of phenotypic variability and the declining reliance on physical examination in delayed recognition. A 27-year-old male with obesity, low libido, and biochemical and clinical primary hypogonadism was found to have 47, XXY karyotype, consistent with KS. His hypogonadism was initially attributed to obesity and overlooked, despite classic signs of a micropenis and small testes. The case highlights the importance of physical examination, comprehensive history, and clinician awareness in diagnosing KS, particularly in atypical presentations. KS is associated with increased risks of osteoporosis, cardiovascular disease, and psychosocial challenges. Raising awareness and focusing on physical examinations can improve diagnostic timing and reduce complications.

## 1. Introduction

We present a case of Klinefelter syndrome (KS) diagnosed in adulthood, highlighting the challenges in recognizing this common yet underdiagnosed condition. Early diagnosis is critical due to the association of KS with metabolic, cardiovascular, and psychosocial comorbidities. This report explores factors contributing to diagnostic delays, including phenotypic variability, limited awareness, and reduced emphasis on physical examination.

## 2. Presentation

A 27-year-old male was referred to endocrinology by his primary care physician (PCP) for low testosterone with fatigue, low libido, and erectile dysfunction. The patient was found to be biochemically hypogonadal after being diagnosed with HIV. The patient reported lifelong low libido, which he had assumed was normal; he is not significantly distressed by sexual dysfunction. He is sometimes able to achieve stimulated erections. Psychiatric history included attention-deficit/hyperactivity disorder (ADHD) diagnosed in childhood and depression diagnosed in adulthood. He had no history of learning disability or behavioral problems beyond ADHD. He reported no testicular trauma, fractures, voice changes, or developmental delays. Puberty occurred at age 13, but he struggled with obesity and sparse hair growth since adolescence. He was diagnosed with HIV in 2023 and has been on antiretroviral therapy (Symtuza 800-150-200-10 mg, a fixed-dose combination of darunavir ethanolate, cobicistat, emtricitabine, and tenofovir alafenamide) with good adherence and no progression of symptoms. Initially, he was started on testosterone gel by his PCP, but he discontinued it due to concerns about transfer risk at work.

Physical examination revealed gynecomastia, nonpalpable testicles, micropenis, and sparse facial and body hair. His height and BMI were 180.34 cm and 51 kg/m^2^, respectively. Karyotyping confirmed a 47, XXY pattern consistent with KS. Biochemical evaluation revealed the following: sex hormone binding globulin (SHBG): 10 (10–50 nmol/L), cortisol: 11.70 (6.02–18.4 µg/dL), ACTH: 18 (6–50 pg/mL), LH: 28.4 (1.7–8.6 mIU/mL), prolactin: 6.8 (4.0–15.2 ng/mL), IGF-1:163 (63–373 ng/mL), IGF-1Z score (male): −0.2 (−2.0−2.0 SD), testosterone: 42 (250–1100 ng/dL), and testosterone free: 8.7 (35–155 pg/mL). Mammogram revealed gynecomastia without evidence of malignancy. Testicular ultrasound showed bilateral small-volume testes: left testicle 2.2 cm × 0.8 cm × 1.2 cm (volume 1.1 cc) and right testicle 1.9 cm × 0.9 cm × 1.4 cm (volume 1.3 cc), with normal echogenicity and no mass. He was started on intramuscular testosterone with reported improvement in mood and energy. A DXA scan was ordered to evaluate bone density.

## 3. Discussion

KS is the most common chromosomal disorder in men, yet it remains significantly underdiagnosed or diagnosed late. It is characterized by a sex chromosome aneuploidy, where one or more extra X chromosomes are present alongside the normal male karyotype of XY. Most individuals with KS have the 47, XXY karyotype, while the remaining 10%–20% exhibit mosaicism [1–3].

Initially described in 1942, KS occurs in approximately 1 in 660 males [1]. Despite its prevalence, KS continues to elude timely diagnosis due to its phenotypic variability. While classic features such as gynecomastia, pubertal arrest, eunuchoid habitus, and small testes may lead to early recognition, atypical presentations often delay diagnosis. Some patients experience an initial increase in testicular size during early puberty, complicating early recognition [1]. Small testes remain the most consistent phenotypic feature across different stages of life, while other characteristics are variably present [2, 3].

The case we present illustrates this diagnostic challenge. Our patient, a 27-year-old male, was referred for evaluation of low testosterone. He lacked classic KS features such as excessive height and overt gynecomastia. Additionally, his elevated BMI masked the diagnosis, as his hypogonadism was initially attributed to obesity rather than KS. Physical examination during his endocrine evaluation revealed nonpalpable testes and micropenis—critical findings that had previously been overlooked. This underscores the critical role of thorough physical examinations in prompting early suspicion and timely referrals to endocrinology [3].

Testicular ultrasound confirmed the presence of bilaterally atrophic testes, consistent with KS. Diagnosis is increasingly made in utero via noninvasive prenatal testing or amniocentesis. However, many remain undiagnosed until adulthood. Clues in childhood or adolescence include learning difficulties, ADHD, behavioral issues, and pubertal abnormalities. Our patient had childhood ADHD and low libido since puberty, but these features were never linked to a syndromic diagnosis.

Importantly, he was diagnosed with KS following HIV-related evaluation. HIV can contribute to both central and primary hypogonadism, though it more commonly causes central hypogonadism. However, our patient had a biochemical pattern of primary hypogonadism, and his symptoms preceded his HIV diagnosis, supporting a KS etiology. Moreover, his antiretroviral regimen (Symtuza) may increase testosterone levels, which necessitates careful monitoring of testosterone response and symptom correlation during replacement therapy.

KS is associated with suboptimal metabolic profiles and an increased risk for osteoporosis, obesity, cardiovascular disease, and certain malignancies [4, 5]. Furthermore, the psychosocial impacts of delayed diagnosis cannot be ignored; these include challenges with self-esteem and social integration [2]. Studies have highlighted both the lack of physician awareness and the declining emphasis on physical examination skills as contributors to diagnostic delays [6]. While adulthood diagnosis falls outside the realm of early detection, raising awareness of KS and emphasizing annual physical examinations, especially by PCPs, school doctors, and sports medicine professionals, could significantly improve diagnostic timing and reduce associated morbidity [1, 2].

In this case, the patient's mild phenotype delayed his diagnosis until adulthood. Despite presenting with symptoms of hypogonadism and ADHD, he was initially managed with testosterone replacement therapy without a comprehensive workup to rule out KS. This highlights the need for heightened clinical awareness among healthcare providers, particularly regarding the spectrum of KS presentations. A more proactive approach to physical examinations and early suspicion of KS, even in patients without overt classic features, could lead to earlier interventions and improved long-term outcomes.

## 4. Conclusion

This case underscores the challenges in diagnosing KS, particularly in atypical presentations. Early recognition through comprehensive evaluations, including physical examination, is essential to mitigate adverse outcomes. Enhanced awareness is key to preventing diagnostic delays.

## Data Availability

Data supporting the findings of this study are available within the article. Further details are available from the corresponding author upon reasonable request.
